# Endophytic Fungi as Pretreatment to Enhance Enzymatic Hydrolysis of Olive Tree Pruning

**DOI:** 10.1155/2017/9727581

**Published:** 2017-11-07

**Authors:** Raquel Martín-Sampedro, Juan Carlos López-Linares, Úrsula Fillat, Guillermo Gea-Izquierdo, David Ibarra, Eulogio Castro, María E. Eugenio

**Affiliations:** ^1^INIA-CIFOR, Ctra de la Coruña, Km 7.5, 28040 Madrid, Spain; ^2^Chemical, Environmental and Materials Engineering Department, Jaén University, Campus Las Lagunillas, s/n, 23071 Jaén, Spain

## Abstract

Olive tree pruning, as one of the most abundant lignocellulosic residues in Mediterranean countries, has been evaluated as a source of sugars for fuel and chemicals production. A mild acid pretreatment has been combined with a fungal pretreatment using either two endophytes (*Ulocladium* sp. and* Hormonema* sp.) or a saprophyte (*Trametes* sp. I-62). The use of endophytes is based on the important role that some of them play during the initial stages of wood decomposition. Without acid treatment, fungal pretreatment with* Ulocladium* sp. provided a nonsignificant enhancement of 4.6% in glucose digestibility, compared to control. When a mild acid hydrolysis was carried out after fungal pretreatments, significant increases in glucose digestibility from 4.9% to 12.0% (compared to control without fungi) were observed for all fungal pretreatments, with maximum values yielded by* Hormonema* sp. However, despite the observed digestibility boost, the total sugar yields (taking into account solid yield) were not significantly increased by the pretreatments. Nevertheless, based on these preliminary improvements in digestibility, this work proves the potential of endophytic fungi to boost the production of sugar from olive tree pruning, which would add an extra value to the bioeconomy of olive crops.

## 1. Introduction

Biorefinery is an overall concept of a sustainable, integrated, and diversified processing plant where biomass feedstocks are converted into a wide range of valuable products (materials, chemicals, and food and feed additives) and energy (fuels, power, and/or heat) [1–3]. Biorefineries can use all kinds of biomass sources, including forestry woody feedstocks and agricultural residues, energy crops, industrial residues, municipal solid wastes, and algae and seaweeds [3]. Among them, the use of lignocellulosic residues such as olive tree pruning is of especial interest not only to revalorize a cheap raw material but also to reduce the problem of its disposal on the fields. Olive biomass after pruning is abundantly generated in Mediterranean countries (3000 Kg/ha/year), and it is mainly composed of a woody fraction and a remaining portion containing leaves and fine branches [4]. Olive tree pruning biomass has been suggested as raw material for a wide range of products [4, 5]. These include products such as bioethanol, oligosaccharides that act as prebiotics, cellulose for paper pulp manufacture [6–8], and others such as polyphenols-based antioxidant and/or extractives for cotton dye manufacture [9, 10].

One of the downsides of the biochemical conversion of lignocellulosic biomass in a biorefinery is the need of an efficient pretreatment that enhances the enzymatic depolymerisation of biomass into fermentable sugars that can be subsequently fermented into fuel and chemical products [11]. This pretreatment step should not only disrupt the complex and recalcitrant lignocellulosic structure making the carbohydrates more accessible to hydrolytic enzymes [12], but also avoid the degradation or loss of sugars and the generation of inhibitory products (e.g., weak acids, furan derivatives, and phenols) that affect the downstream hydrolysis and fermentation steps [13]. Current leading pretreatment technologies are based on physicochemical processes, which in most cases involve high energy demand, high capital costs, partial biomass degradation, and formation of inhibitory byproducts [13]. As an alternative, different biological approaches have been developed as environmentally friendly tools to alter the lignocellulosic structure [14, 15]. In contrast to physicochemical technologies, these biological methods require low capital investment, low energy demand, and milder reaction conditions. Furthermore, these biological processes do not use chemical-based catalysts and do not release inhibitory compounds. However, some of these biological methods require long pretreatment times.

Among biological methods, different wood-decaying fungi have been widely evaluated as a pretreatment to improve the subsequent enzymatic hydrolysis, white-rot basidiomycetes being the most efficient microorganisms for this purpose [14, 15]. Biological pretreatments help to degrade and/or modify lignin, which leads to an increase in the number of pores and the available surface area of pretreated materials and consequently an enhancement in the accessibility of hydrolytic enzymes to sugar fractions [15, 16]. The process includes different enzymatic activities such as peroxidases, laccases, and reductases but also low molecular weight compounds that mediate the action of these enzymes [17]. Basidiomycetes such as* Phanerochaete chrysosporium* [18],* Trametes versicolor* [19],* Ceriporiopsis subvermispora* [20],* Pycnoporus cinnabarinus* [21], and* Panus tigrinus* [19] have shown their potential to pretreat different lignocellulosic feedstocks and enhance sugar yields. Moreover, biological pretreatments have been also combined with other pretreatment methods, including mild acid hydrolysis [22], alkali extraction [19], organosolv [23], and steam explosion [24]. Thus, by combining these pretreatment methods the delignification efficiency can be improved, while the severity conditions, the pretreatment time, and the chemical and energy requirements of nonbiological pretreatment can be reduced [15].

In addition to white-rot basidiomycetes, the capacity of certain endophytic fungi (mainly ascomycetes) to produce some of these ligninolytic enzymes (specially laccases) [25] has also been recently studied. The use of some endophytic fungi as pretreatment step has proved to improve saccharification yields [26], as well as chemical and mechanical pulping [27]. In contrast to white-rot fungi, which act on biomass in an advanced state of degradation, endophytic fungi are involved in the initial stages of biomass decay. These fungi inhabit asymptomatic plant tissues, living in association with their host plants. Once the plant dies, some of these endophytes change from an inactive state to become primary colonizers involved in the decomposition of plant tissues [28], developing complex enzymatic systems and metabolites highly specialized to degrade lignocellulosic substrates of their particular host species.

This study is a continuation of our recent study [26] where we used endophytic fungi* Ulocladium* sp. and* Hormonema* sp., isolated from* Eucalyptus* sp. trees, alone or in combination with an autohydrolysis pretreatment to enhance saccharification of* Eucalyptus globulus* wood. In the present study, the same two fungi are tested as a biological pretreatment to enhance sugar production from olive tree pruning biomass. Combination of biological pretreatment with mild acid hydrolysis is also explored. Chemical composition of resulting pretreated samples and improvements in glucose and xylose digestibility levels are evaluated. The white-rot fungus* Trametes* sp. I-62 is used as reference.

## 2. Materials and Methods

### 2.1. Chemicals, Raw Material, and Enzymes

Reagent-grade chemicals were supplied by Sigma-Aldrich (Madrid, Spain), Merck (Barcelona, Spain), and Panreac (Barcelona, Spain).

Olive tree (*Olea europaea* L.) pruning (OTP) residue was supplied by the Universidad de Jaén (Spain). A typical OTP lot includes leaves (approximately 25% by weight), thin branches (approximately 50% by weight), and thick branches or wood (approximately 25% by weight), although the proportions may vary depending on culture conditions, tree age, production, and/or local pruning practice. This OTP residue was collected after fruit-harvesting. Then it was air-dried at room temperature to equilibrium moisture content (9.1%), milled using a laboratory hammer mill (Retsch) to a particle size smaller than 4 mm, and homogenized and stored until use. The material showed the following composition (% dry weight): ash, 3.4; extractives, 31.4 (of which 7.9 is glucose); cellulose, 22.5; hemicelluloses, 14.2 (xylan, 10.0; galactan 1.3; arabinan, 2.2; mannan, 0.7); Klason lignin, 16.6; acid soluble lignin, 2.2.

The cellulolytic complex Cellic CTec2 was provided by Novozymes (Bagsvaerd, Denmark) (90 FPU/ml cellulase activity). This enzymatic complex was supplemented with Novozyme 188 which mainly contains *β*-glucosidase activity (1274 IU/ml) and was also supplied by Novozymes.

### 2.2. Fungal Strain

*Ulocladium* sp. and* Hormonema* sp. were selected among more than 100 strains of endophytic fungi isolated from* Eucalyptus* sp. trees in Spain, based on their potential to enhance enzymatic hydrolysis of* Eucalyptus globulus* [26]. Details about isolation, screening, and identification of the endophytic fungi can be found in Fillat et al. [25] and Martín-Sampedro et al. [26]. The saprophytic white-rot fungus* Trametes* sp. I-62 was used as reference and was obtained from the collection of Instituto Jaime Ferrán de Microbiología, CIB (Madrid, Spain).

### 2.3. Fungal Pretreatment

Fungi were grown for 7–15 days on 2% malt extract media plates supplemented with 2% agar at 23°C. The content of 1 plate of 90 mm Ø (mycelium plus agarized medium) was used to start a preinoculum in 100 ml of sterilized distilled water. Preinocula were incubated at 23°C on a rotary shaker at 100 rpm for two days and then used to inoculate 100 g (on dry basis) of autoclaved OTP biomass. Consistency was adjusted to 20% by adding sterilized distilled water. Two replicates of inoculated and control samples (without preinoculum) were prepared on 2 L reactors equipped with a system that provides a flow of sterilized wet air into each reactor for 1 min each hour. Reactors were placed in a device rotating at 1.25 rpm for 1 min each hour. After 28 days at 23°C of fungal pretreatment, 650 mL of acetate buffer (pH 5.2) was added to each reactor and stirred at 150 rpm for 1 h to remove superficial mycelium. Then, samples were filtered and OTP weight loss values were calculated from dry weight of biomass before and after pretreatment. Liquid was used to determine enzyme activities (laccase and peroxidase). Solid fraction was stored at 4°C without drying before next treatment. More details about these procedures can be found in Martín-Sampedro et al. [26, 27].

A mild alkali extraction after fungal pretreatment has been reported to improve fungal effect on a subsequent enzymatic hydrolysis [19]. For this reason, all fungal pretreated and control samples were subjected to an alkali extraction at 50°C and 165 rpm for 1 h with a final concentration of 0.1% sodium hydroxide and 5% w/w consistency. Samples were then filtered and washed with distilled water until neutral pH.

### 2.4. Acid Hydrolysis

Each fungal pretreated sample, subjected also to an alkaline extraction, and its respective control sample were divided into two samples. One of them was subjected directly to enzymatic hydrolysis according to Section 2.5. The other sample was subjected to an acid hydrolysis prior to enzymatic hydrolysis. This mild acid hydrolysis was carried out in an autoclave (Trade Raypa S.L., Spain) at 130°C for 60 min, with a liquid-to-solid ratio of 6 : 1 and a final concentration of sulphuric acid of 0.5% (w/w oven dry OTP).

### 2.5. Enzymatic Hydrolysis

Samples resulting from fungal pretreatments (with or without a subsequent acid hydrolysis) as well as their respective control samples were filtered, washed with distilled water, and air-dried at room temperature. Then, they were ground in a Wiley mill and sieved to select a size of 0.25–0.20 mm and subjected to enzymatic hydrolysis. A cellulolytic complex (Cellic CTec 2) supplemented with *β*-glucosidase (Novozym 188) was added to a 5% w/w milled OTP suspension in 50 mM sodium citrate buffer (pH 4.8) to reach a final dose of 15 FPU and 15 IU, respectively, per gram of dry sample. Enzymatic hydrolysis was performed in triplicate at 50°C and 120 rpm for 168 h. Samples of 1.5 mL were taken after 24, 48, 72, and 168 hours of enzymatic hydrolysis to evaluate glucose and xylose concentrations. Hydrolysed samples were heated in boiling water for 10 min to stop the enzymatic reaction, centrifuged at 10000 rpm for 10 min, and filtered through a 0.45 *μ*m nylon syringe filter. Then, samples were analysed by High Pressure Liquid Chromatography (HPLC) using an Agilent Technologies 1260 HPLC fitted with a refractive index detector (Agilent, Waldbronn, Germany) and an Agilent Hi-PlexH column operated at 65°C with a mobile phase containing 5 mmol L^−1^ sulphuric acid pumped at a rate of 0.6 mL min^−1^.

Glucose (*D*_G_) and xylose (*D*_X_) digestibilities were calculated according to (1). Thus, digestibility evaluates the percentage of sugars that were converted during the enzymatic hydrolysis per grams of sugars in the pretreated material (material subjected to enzymatic hydrolysis), as resulting from the chemical analysis (Section 2.6).

(1) DG or DX%=g of sugars in liquid phaseg of sugars in pretreated material×100=Ch×Vhmp×Cp×100,

where *C*_h_ is the concentration of sugars (glucose or xylose) in the hydrolysate at the end of the enzymatic hydrolysis, expressed in g L^−1^; *V*_h_ is the volume of hydrolysate in L; *m*_p_ are the g of dry pretreated material subjected to enzymatic hydrolysis; and *C*_p_ is the fraction of sugars (glucose or xylose) in the pretreated material, expressed as a percentage.

### 2.6. Analytical Methods

The composition of the solid samples obtained after each treatment was determined according to National Renewable Energy Laboratory NREL/TP-510-42618 [29]. High Pressure Liquid Chromatography (HPLC) analyses were performed using an Agilent Technologies 1260 chromatograph fitted with a refractive index detector and an Agilent Hi-PlexPb column (Agilent, Waldbronn, Germany), according to Martín-Sampedro et al. [26].

Glucose and xylose concentrations in the hydrolysates were determined according to Martín-Sampedro et al. [26], using the same HPLC equipment described above fitted with a refractive index detector and an Agilent Hi-PlexH column (Agilent, Waldbronn, Germany). This equipment was also used to quantify sugar and oligomeric contents of the liquid fractions obtained after acid hydrolysis according to NREL/TP-510-42623 [30].

### 2.7. Statistical Analyses

To calculate differences between the two factors applied (i.e., three fungi plus a control and two sequences of treatments, namely, with or without acid hydrolysis before enzymatic hydrolysis) for the different levels of glucose and xylose digestibilities observed after the final enzymatic hydrolysis, we used a linear mixed model [31, 32] because the experiment was sequential; hence data were “a priori” not totally independent. The model expression tested was the following:

(2) yijk=μ+a+αi+βj+γij+εijk,with ε~N0,σ1.

*y*_*ijk*_ are either glucose or xylose digestibility values, with *μ*, *α*_*i*_, *β*_*j*_, and *γ*_*ij*_ being fixed factors, *i* = [1,4] corresponding to four levels of fungi pretreatments (control + three fungi), *j* = [1,2] corresponding to two different sequential treatment types (with or without acid hydrolysis, i.e., acid or nonacid), and *γ*_*ij*_ being the interaction between the two factors. *b* corresponds to an intercept random effect with distribution *b* ~ *N*(0, *σ*_2_). Significance of factors was assessed using analysis of deviance between models differing in specific parameters fit with a maximum likelihood estimator (ML). Model residuals were assessed for normality and homoscedasticity. The final model was fit using a restricted maximum likelihood estimator (REML). All analyses were performed in R [33].

## 3. Results and Discussion

### 3.1. Fungal Pretreatment

After 28 days of fungal pretreatment, weight loss values of 16–22% were observed for the two endophytic and saprophytic fungi assessed (Table S1 in Supplementary Material available online at https://doi.org/10.1155/2017/9727581). This effect is due to fungal degradation of wood components, mainly extractives and some lignin. However, some carbohydrates were also consumed by fungi. These weight loss values were higher than those observed previously when the same endophytic and saprophytic fungi were applied over* Eucalyptus globulus* biomass (1–6%) [26, 27]. Great variability in weight loss upon application of different fungal pretreatments has been described in the literature. For instance, Mardones et al. [34] reported a weight loss of 5.5% during biotreatment of* Eucalyptus nitens* with the white-rot fungus* C. subvermispora*, whereas a range in weight loss between 2% and 35% was described by Cianchetta et al. [35] and Salvachúa et al. [19] depending on the pretreatment duration and the basidiomycete applied using wheat straw as lignocellulosic raw material. Therefore, weight losses depend not only on the fungi applied but also on the type of biomass treated. In the case of OTP biomass, the following should be taken into account: the great amount of water-soluble extractives that this residue contains, 21–31% [36–40], because it includes not only a woody fraction but also leaves and fine branches. Part of these water-soluble extractives could be partially removed by the distilled water used during the 28 days of fungal pretreatment and the acetate buffer added at the end of the treatment before filtration, as confirmed by the weight loss observed in control samples: 5.5% (Table S1, Supplementary Material). The sugar composition of the liquid fraction collected after the control pretreatment also corroborates this fact (Table 1), as it included a high amount of sugars, mainly glucose in monomeric and oligomeric forms, and arabinose. This is due to the presence of nonstructural sugars such as oleuropein, a glucoside present in the leaves [41], that is readily soluble and can be easily recovered in water extraction process [42]. A significant reduction in sugar content was found when comparing the composition of this control liquid fraction with that obtained after any fungal pretreatment, which indicates that fungi consumed these readily accessible sugars during the pretreatment.

Regarding enzymatic activities at the end of the fungal pretreatments, 16.6 IU g^−1^ (over dry weight) of laccase activity was detected in sample pretreated with* Trametes* sp. I-62, whereas no significant peroxidase or laccase activities were found in the other samples. Nevertheless, since activity was only measured at the end of the fungal treatment, these results do not imply that endophytic fungi did not produce ligninolytic enzymes along the fungal treatment. For instance, a significant amount of ligninolytic activities (mainly laccase activity) was detected after 28 days of fungal treatment when these endophytic fungi were applied over* E. globulus* biomass, while neither cellulase (endo- or exoglucanase) nor xylanase activities were found [26, 27].

After fungal pretreatment, all resulting samples were subjected to a mild alkali extraction in order to improve their digestibility without masking fungal effect on a subsequent enzymatic hydrolysis [19]. As it can be observed in Table 1, the liquid fraction collected from this extraction did not contain a significant amount of sugars or degradation products (including not only those compounds shown in Table 1 such as acetic acid, furfural, and HMF, but also formic acid, levulinic acid, mannitol, and xylitol, which were also determined although data are not shown) confirming that this alkaline extraction is a mild treatment that does not affect in a significant way the sugar composition of the fungal pretreated biomass. However, a 14-15% lignin was extracted during this extraction in samples biopretreated with* Hormonema* sp. and* Trametes* sp. I-62 compared to a 2% in control and* Ulocladium* sp. biopretreated samples (Table 2 and Table S1, Supplementary Material). This lignin extraction was probably explained by the extraction of phenol compounds (solubles in alkali conditions) coming from the modification of lignin by fungi. The removal of these inhibitory compounds could improve the subsequent enzymatic hydrolysis, explaining the results found by Salvachúa et al. (2011) which reported that digestibility increased more than twice in several biopretreated samples when this mild alkali extraction was applied.

### 3.2. Acid Hydrolysis

**Figure 1:**
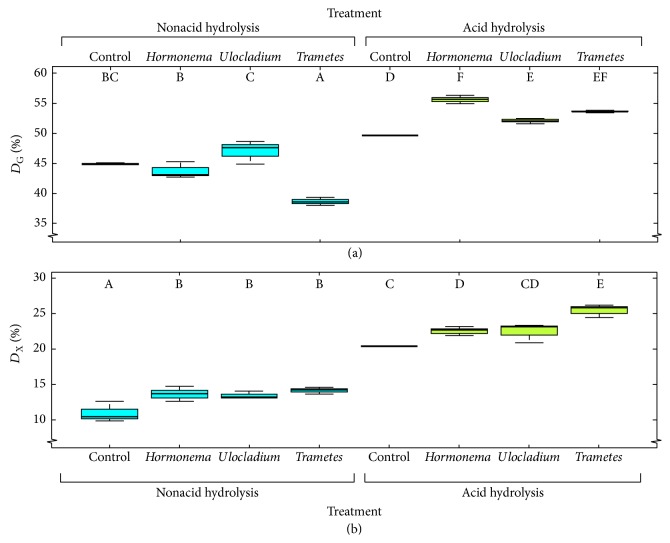
(a) Glucose digestibility (*D*_G_) and (b) xylose digestibility (*D*_X_) achieved after 168 h of enzymatic hydrolysis of OTP samples subjected to fungal pretreatment (followed by alkali extraction) and a subsequent, or not, acid hydrolysis (acid hydrolysis and nonacid hydrolysis, resp.). Means were calculated from three replicates within each group. Different letters represent significant differences at *α* > 0.05 using contrasts and the best models from Tables 3 and 4.

An acid hydrolysis was applied on the samples resulting from the different fungal pretreatments (followed by alkaline extraction) to evaluate the effect of this treatment on saccharification yields. A low severity was selected (concentration of 0.5% H_2_SO_4_ and severity factor of *S*_0_ = 2.66, calculated according to Overend et al. [43]) in order to improve the saccharification process without masking the influence of each fungus as a result of a more aggressive hydrothermal treatment. As a result of these selected conditions for acid hydrolysis, digestibility values of treated samples were still low (Figure 1, as it will be discussed in Section 3.3). Nevertheless, once the most adequate fungus was selected, a more intensive treatment could be carried out in order to maximize sugar recovery (taking into account both the sugars loss on the treatment and the sugars yield on the enzymatic hydrolysis).

Table 2 shows the composition of the samples resulting from fungal pretreatment (followed by mild alkali extraction) and a subsequent, or not, acid hydrolysis. Samples that were not subjected to acid hydrolysis exhibited a slight increase in Klason lignin compared to control samples. This was probably due to the removal of other components such as water-soluble extractives and nonstructural sugars, which are abundant in OTP, as mentioned above. Although these compounds were removed also in control samples (Table 1), fungal pretreatments increased the accessibility of biomass, making the sugar extraction more evident. However, these compounds are not detected on the liquid fraction (Table 1) because fungi use them as carbon sources. On the other hand, glucan and xylan contents were similar in most nonacid-hydrolysed samples, except for samples biopretreated with endophytic fungi, which showed slightly lower xylan content (Table 2). The tested fungi were not selective for holocellulose or lignin in presence of this high amount of water-soluble extractives.

Regarding samples subjected to acid hydrolysis, all of them showed a similar composition, except for a reduction of ethanol-soluble extractives in fungal treated samples compared to control, especially in sample biopretreated with* Ulocladium* sp. Slightly higher glucan and xylan content values were also observed in sample biopretreated with* Trametes* sp. I-62.

When the composition of nonhydrolysed and acid-hydrolysed samples were compared taking into account also the weight losses (Table S1, Supplementary Material), the effect of the acid hydrolysis could be quantified in terms of extraction of each wood component. 27% of ethanol-soluble extractives contained in the nonhydrolysed sample were removed in control samples during acid hydrolysis, compared to a 44%, 56%, and 25% observed in samples biopretreated with* Hormonema* sp.,* Ulocladium* sp., and* Trametes* sp. I-62, respectively. These data seem to indicate that endophytic fungi are more effective in degrading some wood or leave components that became soluble in a subsequent acid hydrolysis treatment.

Klason lignin content (%) increased in acid-hydrolysed samples, compared to content in samples before acid hydrolysis treatment, which could be partially due to the removal of other components during this chemical treatment (Table 2). However, these increases in Klason lignin content were much greater than they should have been just taking into account the solubilisation of other components. Thus, on the basis of solubilisation (according to the solid recovery yield and acid-insoluble lignin) Klason lignin content up to 24.4%, 26.2%, 25.1%, and 26.3% was expected for control,* Hormonema* sp.,* Ulocladium* sp., and* Trametes* sp. samples, respectively. Other authors observed similar or even higher increases in Klason lignin when OTP biomass was subjected to hydrothermal treatments (autohydrolysis, steam explosion, or acid dilute treatments) [6, 38, 44, 45]. This fact could be explained by the formation of “lignin-like” structures as a result of condensation reactions between lignin and carbohydrate degradation products or extractives. These structures are insoluble in acid and therefore determined together with true lignin in the procedure to analyse Klason lignin content. In the case of OTP biomass, which contains not only a woody fraction but also leaves, this effect is especially important, due to its high extractive contents. Flavonoids as luteinin and apigenin have been described in olive leaves and may be involved in the formation of these condensed molecules during hydrothermal treatments by participating in condensation reactions with sugar degradation products [45]. According to Cara et al. [38], when OTP was subjected to a preextraction before hydrothermal treatment, acid-insoluble lignin was significantly lower than that measured on the unextracted material, supporting the idea that the quantification of Klason lignin can be interfered by the presence of high extractives content.

Although glucan content was similar in all samples (Table 2), if solid recovery yield was taken into account (Table S1, Supplementary Material), 4–19% of the glucan contained before acid hydrolysis was estimated to be extracted during acid hydrolysis, being more significant in control samples. The analysis of the liquid fraction obtained (Table 1) also corroborates this unusual high extraction of glucose during a mild acid treatment. However, most of the solubilised glucose did not have its origin in cellulose but on starch and glucosides present in the leaves [42, 46, 47]. This nonstructural glucose present in the extractives is released even at mild pretreatment conditions [39, 44].

Regarding hemicellulose extraction, between 8% and 19% of the xylan was extracted during acid hydrolysis (except for sample biopretreated with* Ulocladium* sp.) while almost all the arabinan (75–88%) was removed from all samples (Table 2 and Table S1, Supplementary Material). These data are in agreement with liquid fraction compositions (Table 1), in which glucose and arabinose (mainly in oligomeric forms) are the most abundant components. The low xylose recovery is explained by the low severity factor (*S*_0_ = 2.66) and the low acid concentration (0.5% H_2_SO_4_) selected for this treatment. Increasing both factors, a significant increase in xylose extraction is expected [44, 46]. The high extraction of arabinose is explained by its more labile nature and its presence in ramifications of hemicellulose rather than in the backbone [47].

### 3.3. Enzymatic Hydrolysis

Figure 1 displays glucose and xylose digestibility values for samples after a fungal pretreatment and a subsequent, or not, acid hydrolysis, as well as their respective control samples. Tables 3 and 4 show statistical model results for glucose and xylose digestibility, respectively. The evolution of sugar production during enzymatic hydrolysis can be found in Figure S1 in Supplementary Material, expressed as g of sugar per g of hydrolysed material.

As it can be observed in Figure 1, the highest glucose digestibility without acid hydrolysis was obtained for samples biopretreated with* Ulocladium* sp., which yielded an increase of 4.6% with respect to control sample without fungus. Yet, this increase was nonsignificant. Fungal pretreatment has been reported to enhance enzymatic hydrolysis by breaking lignin-carbohydrate complex [48] and improving porosity of plant material, leading to an increase in the initial adsorption of cellulolytic enzymes to cellulose [49]. Moreover, biological pretreatments can produce a lignin modification (hydroxylation, demethoxylation, and shortening of side chains) that leads to reducing the unproductive binding of hydrolytic enzymes and, consequently, to enhancing the saccharification process [16]. However, without an acid hydrolysis no improvements in digestibility values were found in samples biopretreated with the other fungi evaluated. Moreover, even a decrease in glucose digestibility was observed in samples biopretreated with* Trametes* sp. I-62 compared to control, contrary to what was expected. In this sense, higher saccharification enhancements were observed when the same fungi were applied to* E. globulus* chips [26], especially for the saprophytic fungus (i.e.,* Trametes* sp.). In this cited work, it was observed that endophytic fungi were able to act in both untreated and autohydrolysed* E. globulus*, without any differences in hydrolysis yields when the sequential order of the pretreatments (fungal and autohydrolysis pretreatments) was altered [26]. However, those samples pretreated with* Trametes* sp. I-62 showed lower saccharification yields when the fungal pretreatment was carried out before autohydrolysis treatments. This indicates that this saprophytic fungus requires a pretreatment which improves accessibility of the material and removes some wood compounds such as extractives and hemicellulose [26]. These data could explain the low glucose digestibility found for OTP samples biopretreated with* Trametes* sp. I-62, especially taking into account the high water extractive content of OTP biomass (21–31%). Furthermore, although endophytic fungi were able to act on untreated* E. globulus* chips, the much higher water-soluble extractives content of OTP biomass could hinder the fungal action.

However, compared to the previously discussed results, glucose digestibility was significantly higher when an acid hydrolysis was carried out on samples with fungal pretreatment, as expected. Acid hydrolysis removes extractives and hemicellulose, increasing the accessible surface area of cellulose [50–52] and therefore the glucose digestibility. Thus, control samples increased its glucose digestibility from 45% to 50% by adding this mild acid hydrolysis treatment. Similar or even lower glucose digestibilities were found by Cara et al. [6] who reported values of 15.3–56.5% when they subjected OTP biomass to a diluted acid pretreatment at 170°C for 10 min (*S*_0_ = 3.06) with sulphuric acid concentration varying from 0.2 to 1.4% followed by 72 h enzymatic hydrolysis. When these authors increased the temperature of the diluted acid treatment (up to 210°C) they achieved digestibilities up to 76.5%. Similarly, Martínez-Patiño et al. [36] found glucose digestibility of 27.8% when OTP was subjected to hydrothermal treatment of similar severity to (but higher temperature than) the one performed in our work (160°C, 10 min, *S*_0_ = 2.77) without the addition of acid. These authors obtained higher digestibilities by increasing acid concentration to 4% and 8% (digestibility increase to 61–70.2%) and/or increasing treatment temperature up to 200°C (digestibility increase to 97.7% in the most severe conditions of temperature and acid concentration). When phosphoric acid was used instead of sulphuric acid, Martínez-Patiño et al. [37] reported a digestibility of 59.4% after a pretreatment at *S*_0_ = 3.06 (170°C, 10 min) and 0.5% phosphoric acid concentration, which increased up to 85.8 by increasing temperature (up to 210°C) and acid concentration (up to 1.5%). Therefore, if the interest is to optimize the sugar recovery after enzymatic hydrolysis, higher temperature and/or acid concentration should be used. However, the objective of our study was to evaluate the effect of endophytic fungi as pretreatment and its combination with acid hydrolysis without masking the influence of each fungus as a result of more aggressive hydrothermal treatment, as mentioned above.

Therefore, differences in glucose digestibility between fungi mostly varied between the two sequences applied (fungal pretreatment and a subsequent, or not, acid hydrolysis), as reflected by the significant interaction between factors (Table 3). Thus, when an acid hydrolysis was performed, all fungi enhanced saccharification compared to control sample with acid hydrolysis (glucose digestibility of 49.7%). In this sequential treatment, the highest glucose digestibility (55.6%) was found for samples biopretreated with* Hormonema* sp. which was able to enhance saccharification by 12.0% compared to control, while* Trametes* sp. I-62 achieved an increase of 8% compared to control. These data prove the potential of this endophytic fungus to boost saccharification not only of* E. globulus*, as it was previously reported [26, 53, 54], but also of OTP biomass. However, total sugar yields (g per 100 g of initial untreated OTP biomass) were not significantly increased compared to untreated control (Table 5).

Several authors have reported the inhibition of enzymatic hydrolysis depending on the lignin content and its distribution on plant material, not only by physically limiting accessibility to cellulose but also by reversible/irreversible adsorption of hydrolytic enzymes onto lignin [23, 28, 55–57]. However, no relation was found when comparing lignin content and glucose digestibilities in the present work. This finding is in agreement with those of several other authors [19, 26, 35] who observed that although lignin attack is essential to the efficiency of the enzymatic hydrolysis of cell wall polysaccharides, the highest lignin degradation is not always positively correlated with the highest levels of cellulose and hemicellulose digestibility. Thus, fungal pretreatment can produce lignin modifications (hydroxylation, demethoxylation, and shortening of side chains) without reducing lignin content, boosting enzymatic hydrolysis due to a reduction of unproductive binding of hydrolytic enzymes onto lignin [16].

The efficient use of all sugars present in lignocellulosic materials is crucial to increase the profitability of biorefineries. Since xylose is the second most abundant carbohydrate in nature, its transformation into fuel and chemical products by biological methods (e.g., fermentation processes using novel fermenting microorganisms with the capacity to convert all kinds of sugars) is essential to improve the global economy of the process. In this context, xylose digestibility of OTP biomass subjected to the different fungal and chemical treatments was also studied herein. Then, all fungal pretreatments followed or not by a subsequent acid hydrolysis improved xylose digestibility by 10–30% compared to control. When an acid hydrolysis was added, xylose digestibility was enhanced, although the xylan content of the treated material was lower limiting the xylose concentration in the hydrolysate. Similarly to that for glucose and as reflected by the significant interaction between the two factors (Table 4), differences between fungi also varied between the two different sequences applied (with or without acid hydrolysis). While no significant differences between the different fungal pretreatments were observed without acid hydrolysis,* Trametes* sp. I-62 provided higher enhancements than endophytic fungi. Then, when an acid hydrolysis was carried out, there was an increase in xylose digestibility from 20.4% for control to 22.6% and 22.5% for* Hormonema* sp. and* Ulocladium* sp., respectively, and to 25.5% for* Trametes* sp. Finally, the random effect taking into account the possible correlations among observations was not significant for glucose and xylose digestibilities. This means that in fact the data could have been analysed as a general linear model just including the two factors and their interaction (Tables 3 and 4), with identical results to those reported here.

## 4. Conclusions

Fungal pretreatments have been applied for the first time to enhance enzymatic hydrolysis of OTP biomass, aiming to increase valorization of this biomass as sugar source. Moreover, the effects of two endophytic fungi were evaluated and compared to the saprophytic fungus* Trametes* sp. I-62, proving the potential of these two endophytic fungi to pretreat OTP biomass. The highest improvement in glucose digestibility was found when* Hormonema* sp. pretreatment was followed by an alkaline extraction and a subsequent mild acid hydrolysis. However, when no acid hydrolysis was performed, nonsignificant increases in enzymatic hydrolysis yields were observed.

Nevertheless, despite the observed boost in digestibility, the total sugar yields were not significantly increased by the combination of fungal and chemical pretreatments. Furthermore, the high water-soluble extractives content of OTP biomass could have hindered the fungal effectiveness. Therefore, although this work proves the potential of endophytic fungi to enhance sugar digestibility, further researches are needed to identify more effective strategies, in order to obtain higher saccharification yields for a real economical valorization of olive tree pruning.

## Supplementary Material

Supplementary Material file includes weight losses during fungal, alkali extraction and acid hydrolysis pretreatments (Table S1), and evolution of sugar production during enzymatic hydrolysis (Figure S1).

## Figures and Tables

**Table 1:** Composition of liquid fractions collected after fungal pretreatment, alkali extraction, and acid hydrolysis expressed in g L^−1^. Consistency of fungal, mild alkali, and acid hydrolysis treatments was 20%, 5%, and 17%, respectively. Glc: glucose; Xyl: xylose; Ara: arabinose; AcH: acetic acid; GOS: glucooligosaccharides; XOS: xylooligosaccharides; AOS: arabinooligosaccharides; HMF: hydroxymethyl furfural; Furf: furfural.

Sample	Glc	Xyl	Ara	AcH	GOS	XOS	AOS	HMF	Furf
Fungal treatment									
Control	4.12	1.50	7.58	2.95	3.03	0.56	0.18	0.00	0.00
*Hormonema* sp.	0.98	2.48	6.80	2.44	1.50	0.53	0.42	0.00	0.00
*Ulocladium* sp.	1.02	0.54	0.00	2.71	1.96	0.44	0.00	0.05	0.00
*Trametes* sp.	0.46	0.55	4.26	2.60	1.76	0.00	0.00	0.08	0.00
Mild alkali treatment									
Control	0.23	0.16	0.41	0.02	0.16	0.00	0.00	0.00	0.00
*Hormonema* sp.	0.08	0.19	0.53	0.03	0.10	0.01	0.02	0.00	0.00
*Ulocladium* sp.	0.06	0.06	0.00	0.00	0.09	0.02	0.00	0.00	0.00
*Trametes* sp.	0.02	0.04	0.10	0.00	0.13	0.00	0.00	0.00	0.00
Acid hydrolysis treatment									
Control	1.10	0.13	1.78	0.31	7.40	2.50	3.95	0.04	0.03
*Hormonema* sp.	0.98	0.16	1.02	0.22	4.11	1.73	4.42	0.01	0.01
*Ulocladium* sp.	0.95	0.09	0.59	0.18	3.51	1.35	2.20	0.01	0.00
*Trametes* sp.	0.91	0.20	1.69	0.29	2.09	2.48	2.06	0.01	0.01

**Table 2:** Composition of solid fractions obtained after fungal pretreatment (followed by alkali extraction) and a subsequent, or not, acid hydrolysis (acid hydrolysis and nonacid hydrolysis, resp.). Data are expressed in % (w/w).

Sample	Ethanol extractives	Klason lignin	Acid soluble lignin	Total lignin	Glucan	Xylan	Arabinan
Nonacid hydrolysis							
Control	4.6 ± 0.2	18.9 ± 0.1	4.2 ± 0.0	23.1 ± 0.1	31.3 ± 0.4	16.9 ± 0.6	3.6 ± 0.4
*Hormonema *sp.	4.8 ± 0.1	21.2 ± 0.8	4.2 ± 0.0	25.4 ± 0.8	29.6 ± 0.5	15.5 ± 0.3	3.4 ± 0.0
*Ulocladium *sp.	5.0 ± 0.2	21.8 ± 0.1	4.0 ± 0.0	25.9 ± 0.1	30.9 ± 0.8	15.5 ± 0.6	2.3 ± 0.1
*Trametes *sp.	4.0 ± 0.2	21.2 ± 0.1	4.3 ± 0.0	25.5 ± 0.1	29.9 ± 0.1	17.2 ± 0.1	2.2 ± 0.1
Acid hydrolysis							
Control	4.1 ± 0.1	26.6 ± 0.2	4.0 ± 0.0	30.6 ± 0.2	31.1 ± 0.3	16.8 ± 0.2	0.5 ± 0.0
*Hormonema *sp.	3.2 ± 0.2	26.6 ± 0.4	3.9 ± 0.0	30.5 ± 0.4	31.0 ± 0.4	17.0 ± 0.3	0.7 ± 0.2
*Ulocladium *sp.	2.5 ± 0.2	26.3 ± 0.1	4.1 ± 0.0	30.4 ± 0.1	30.6 ± 0.6	17.6 ± 0.1	0.7 ± 0.1
*Trametes *sp.	3.5 ± 0.2	26.1 ± 0.6	3.6 ± 0.0	29.7 ± 0.6	33.8 ± 0.6	18.6 ± 0.3	0.3 ± 0.1

**Table 3:** Model results for glucose digestibility (*D*_G_) and differences between factors tested. Factors = factors included in model *i*; # = number of parameters in model *i*; hydrol = sequential treatment type (with or without acid hydrolysis); *p* values for model *i* were calculated using analysis of deviance compared to a *χ*^2^ distribution calculated between model *i* and model *i* − 1.

Model	Factors	#	Deviance	*p* value	AIC
(1)	—	3	148.6	—	154.6
(2)	Hydrol	4	117.1	<0.001	125.1
(3)	Fungi + hydrol	7	108.5	0.037	122.7
(4)	Hydrol + fungi + hydrol × fungi	10	53.4	<0.001	73.4
(5)	Model M4 without random effect	9	53.4	0.999	71.4

**Table 4:** Model (see (2)) results for xylose digestibility and differences between factors tested. Factors = factors included in model *i*; # = number of parameters in model *i*; hydrol = sequential treatment type (with or without acid hydrolysis); *p* values for model *i* were calculated using analysis of deviance compared to a *χ*^2^ distribution calculated between model *i* and model *i* − 1.

Model	Factors	#	Deviance	*p* value	AIC
(1)	—	3	146.8	—	152.8
(2)	Hydrol	4	88.0	<0.001	96.0
(3)	Fungi + hydrol	7	62.7	<0.001	76.7
(4)	Hydrol + fungi + hydrol × fungi	10	54.1	0.034	74.1
(5)	Model M4 without random effect	9	54.1	0.999	72.1

**Table 5:** Global solid recovery yield of all sequential treatments (i.e., nonacid hydrolysis: fungal treatment + alkali extraction; acid hydrolysis: fungal treatment + alkali extraction + acid hydrolysis) and total sugar yield reported as g of solid material (solid yield) or g of sugars (sugar yield) per 100 g of initial untreated OTP biomass.

Sample	Pretreatments global yield	Total sugar yield
Nonacid hydrol		
Control	89.8 ± 0.4	14.4 ± 0.2
*Hormonema* sp.	80.9 ± 0.3	12.2 ± 0.4
*Ulocladium* sp.	75.2 ± 0.4	12.5 ± 0.5
*Trametes* sp.	75.3 ± 0.3	10.5 ± 0.2
Acid hydrolysis		
Control	72.9 ± 0.5	13.8 ± 0.0
*Hormonema* sp.	68.2 ± 0.6	13.6 ± 0.1
*Ulocladium *sp.	66.5 ± 0.5	13.0 ± 0.2
*Trametes* sp.	64.2 ± 0.4	13.1 ± 0.1
