# Supplementary material for: Endophytic Fungi as Pretreatment to Enhance Enzymatic Hydrolysis of Olive Tree Pruning
Source: Biomed Res Int. 2017 Nov 7;2017:9727581. doi: 10.1155/2017/9727581 (PMC5698607; doi:10.1155/2017/9727581)
Supplement: Supplementary file 1 — Supplementary Material file includes weight losses during fungal, alkali extraction and acid hydrolysis pretreatments (Table S1), and evolution of sugar production during enzymatic hydrolysis (Figure S1). [file 9727581.f1.docx]

**SUPPLEMENTARY INFORMATION**

**Endophytic fungi as pretreatment to enhance enzymatic hydrolysis of olive tree pruning**

Raquel Martín-Sampedro^1,*^, Juan Carlos López-Linares^2^, Úrsula Fillat^1^, Guillermo Gea-Izquierdo^1^, David Ibarra^1^, Eulogio Castro^2^, María Eugenia Eugenio^1^

*^1^INIA-CIFOR, Ctra de La Coruña, Km 7.5 Madrid 28040, Spain;*

*^2^Jaén University. Chemical, Environmental and Materials Engineering Department. Campus Las Lagunillas s/n Jaén 23071, Spain.*

*Correspondence: martin.raquel@inia.es; Tel.: +34-913476834

The following document is complementary to the work presented in the main contribution. It includes weight losses during fungal, alkali extraction and acid hydrolysis pretreatments (Table S1), and evolution of sugar production during enzymatic hydrolysis (Figure S1)

**Table S1**. Weight losses (reported as g per 100 g of solid treated) after fungal treatment, alkali extraction and acid hydrolysis.

| **Sample** | **Fungal treatment** | **Alkali extraction** | **Acid hydrolysis** |
| --- | --- | --- | --- |
| **Control** | 5.5 ± 0.2 | 5.0 ± 0.1 | 18.9 ± 0.2 |
| ***Hormonema* sp.** | 15.8 ± 0.3 | 3.9 ± 0.1 | 15.6 ± 0.4 |
| ***Ulocladium* sp.** | 22.3 ± 0.3 | 3.3 ± 0.2 | 11.5 ± 0.1 |
| ***Trametes* sp.** | 17.7 ± 0.2 | 8.6 ± 0.3 | 14.7 ± 0.2 |

**Figure S1.** Evolution of glucose (a), xylose (b) and total sugar (c) production during enzymatic hydrolysis of the OTP samples subjected to fungal pretreatment (followed by alkali extraction) and a subsequent, or not, acid hydrolysis (acid and non-acid, respectively). Data are reported as g of sugar per g of hydrolysed material, and are means of three replicates.
